# Dose or time? Comparing early prediction of intermittent theta-burst stimulation response across accelerated and daily protocols for depression

**DOI:** 10.1007/s00406-026-02266-2

**Published:** 2026-06-01

**Authors:** Paulo J. C. Suen, Beatriz A. Cavendish, Mariana P. Batista, Ives Cavalcante Passos, Kallene Summer Moreira Vidal, André R. Brunoni

**Affiliations:** 1 Service of Interdisciplinary Neuromodulation, Department, Institute of Psychiatry, University of São Paulo Medical School, São Paulo, Brazil; 2 Laboratory of Neuroscience, National Institute of Biomarkers in Psychiatry, Department and Institute of Psychiatry, University of São Paulo Medical School, São Paulo, Brazil; 3 Graduate Program in Psychiatry and Behavioral Sciences, Department of Psychiatry, School of Medicine, Universidade Federal do Rio Grande do Sul, Porto Alegre, RS Brazil; 4 Division of Interventional Psychiatry, Department of Psychiatry and Peter O’Donnell Brain Institute, UT Southwestern Medical Center, Dallas, TX USA; 5 Department of Psychiatry, UT Southwestern Medical Center, 1440 Empire Central Dr. LD4.246, Dallas, TX 75247 USA

**Keywords:** Major depressive disorder, Theta-burst stimulation, Transcranial magnetic stimulation, Treatment prediction, Accelerated protocols

## Abstract

**Objective:**

To compare early response prediction across accelerated and standard intermittent theta-burst stimulation (iTBS) protocols for major depressive disorder, examining whether predictive performance differs when assessments are matched by cumulative pulse exposure versus calendar time.

**Methods:**

Pooled analysis of two prospective iTBS trials conducted at Instituto de Psiquiatria, Hospital das Clínicas, Universidade de São Paulo: the open-label BRAEN-MAP trial (once-daily; *N* = 65; 20 sessions over 4 weeks) and the active arm of the triple-blind, sham-controlled randomized TTT trial (accelerated; *N* = 50; 45 sessions over 3 weeks). Assessments were matched by cumulative pulses (18,000 pulses), calendar time (Weeks 1 and 2), or session count (15 sessions). Early improvement was defined as ≥ 20% HDRS-17 reduction. Positive predictive values (PPV) and negative predictive values (NPV) were compared using bootstrap resampling.

**Results:**

Response rates did not differ between trials (BRAEN-MAP: 55.4%; TTT: 54.0%; *p* = 0.95). For response prediction, PPV ranged from 66.7% to 75.0% across protocol-timepoint combinations, with no significant differences between protocols at pulse-matched (66.7% vs. 68.9%, *p* = 0.84), time-matched, or session-matched (66.7% vs. 68.8%, *p* = 0.86) comparisons (all bootstrap *p* > 0.43). NPV for non-response prediction ranged from 69.0% to 82.4%, also without significant between-protocol differences. Trajectory analyses demonstrated that early improvers maintained steeper improvement through endpoint.

**Conclusion:**

Early symptom improvement predicts iTBS response consistently across accelerated and standard protocols regardless of whether assessments are matched by cumulative pulses, calendar time, or number of sessions. These preliminary findings support early response assessment as a protocol-independent clinical tool, though the accuracy levels observed (PPV 67–75%) are insufficient to justify treatment discontinuation and may be most useful within adaptive, measurement-based care frameworks.

**Supplementary Information:**

The online version contains supplementary material available at 10.1007/s00406-026-02266-2.

## Introduction

Major depressive disorder (MDD) is a common, chronic, and recurrent condition [1]. Approximately, only 30% of patients achieve remission after initial treatment, and up to 30% remain symptomatic after more than two medication trials [2]. Repetitive transcranial magnetic stimulation (rTMS), which uses electromagnetic pulses to change cortical activity and excitability [3], can alleviate depressive symptoms by targeting and restoring aberrant activity in brain regions implicated in acute depressive episodes, such as the left dorsolateral prefrontal cortex (DLPFC) and its associated networks [4]. Intermittent theta-burst stimulation (iTBS) - a more recently developed patterned form of TMS - was shown to be non-inferior to the standard high-frequency rTMS for treating depression, while substantially reducing session duration [5].

Identifying early markers of treatment response could help optimize both standard and accelerated protocols. In standard once-daily rTMS protocols, early symptom improvement, typically assessed after 5–10 sessions (corresponding to 1–2 weeks of daily treatment), has been investigated as a predictor of clinical response [6, 7]. Several studies reported that patients achieving less than 20% symptom reduction after 10 sessions (2 weeks) were unlikely to respond by the end of treatment, with negative predictive values exceeding 89% [6, 8]. Mirman et al. (2022) further showed that in patients with severe baseline depression, less than 20% improvement in mood symptoms after just 5 sessions (1 week) predicted nonresponse with over 90% certainty [8]. The strength of these predictions, however, appears to depend on treatment course length. In a large registry study (*N* = 7,215), Sackeim et al. (2024) found that the predictive value of early lack of improvement was substantially lower than previously reported when patients completed a full 36-session course: after 10 sessions, accuracy in predicting final nonresponse was only ~ 51%, with large proportions of patients showing minimal early improvement ultimately achieving response [9]. The authors concluded that early symptom change more accurately predicts who will respond than who will not, and that the predictive value of early nonresponse may be insufficient to reliably guide treatment modifications.

However, all of these studies employed once-daily protocols, meaning that session number and elapsed time were inherently confounded. Moreover, recent meta-analytic evidence suggests that the number of sessions, rather than total pulse count, may be the more relevant predictor of rTMS outcome [10], yet in daily protocols, sessions and time cannot be dissociated. This leaves open the question of whether the predictive value of early clinical change is driven by cumulative stimulation dose, number of sessions, the passage of time allowing for neuroplastic consolidation, or a composite of these. Accelerated iTBS (aiTBS) protocols, which deliver multiple sessions per day, offer both a clinical advance and a natural framework to disentangle these parameters. In daily protocols, the first week represents only 5 treatment sessions, a dose generally considered insufficient for reliable outcome prediction [8, 11]; in an accelerated protocol, the same calendar time may comprise 10 or more sessions with substantially higher cumulative pulse exposure. Understanding whether early prediction is consistent across delivery schedules has direct clinical implications, as it would determine whether clinicians can apply the same adaptive decision framework, such as protocol modification for early non-improvers [12], across both standard and accelerated settings.

Therefore, the goal of this study is to compare early symptom trajectories and their predictive utility across accelerated and standard iTBS protocols. Using data from two clinical trials - an accelerated (TTT trial) and a standard once-daily protocol (BRAEN-MAP trial) - we examined whether early improvement predicts endpoint response across protocols when matched by cumulative pulse exposure, calendar time or number of sessions. We hypothesize that early response prediction is consistent across protocols when accounting for cumulative stimulation dose, supporting dose-dependent rather than time- or session-dependent mechanisms of prediction.

## Methods

### Study design

This study reports pooled analyses from two prospective trials of iTBS for MDD conducted at the Instituto de Psiquiatria, Hospital das Clínicas, Universidade de São Paulo, Brazil: (1) the Brazil-England Study on Mechanisms and Response Predictors of iTBS Treatment (BRAEN-MAP; once-daily protocol), an open-label trial conducted between August 2021 and October 2022; and (2) a post-hoc analysis of the active treatment arm from the Triple TBS Thirty-minute Interval (TTT; accelerated protocol) trial, a triple-blinded, sham-controlled randomized clinical trial conducted between July 2022 and June 2024, whose primary results have been published elsewhere [13]. For BRAEN-MAP, this represents the first report of clinical outcomes from this open-label trial. Both studies received approval from institutional and national ethics committees and were registered at ClinicalTrials.gov (BRAEN-MAP: NCT04969549; TTT: NCT05385055).

### Participants

Both trials enrolled adults aged 18–65 years with a MDD diagnosis confirmed by the Mini International Neuropsychiatric Interview (MINI). BRAEN-MAP included participants with baseline Hamilton Depression Rating Scale 17-item version (HDRS-17) score ≥ 14, while TTT required treatment-resistant depression (TRD), defined as failure to respond to at least one but no more than three adequate antidepressant trials in the current episode, and baseline HDRS-17 score ≥ 17. Key exclusion criteria were: primary psychiatric disorders other than MDD (comorbid anxiety disorders were permitted); active suicidal ideation; and contraindications to TMS or magnetic resonance imaging. Participants taking antidepressant medications were required to maintain stable doses for at least 6 weeks prior to enrollment and throughout the trial. Benzodiazepine use was permitted up to 20 mg/day diazepam-equivalent. For the present analysis, we included only the participants randomized to active iTBS treatment from TTT, to compare with the standard daily protocol used in BRAEN-MAP.

### Intervention

Stimulation was administered using a MagPro X100 device with a figure-of-eight B65 coil (MagVenture, Farum, Denmark), with the coil positioned over the left dorsolateral prefrontal cortex (DLPFC) using the modified Beam F3 method [14, 15]. Both trials employed an iTBS pattern consisting of 50 Hz triple-pulse bursts repeated at 5 Hz, with 2 s of stimulation followed by 8 s of rest, and stimulation intensity set at 100% of each participant’s resting motor threshold (rMT). The key protocol differences were: BRAEN-MAP delivered once-daily sessions over 4 weeks (20 total sessions), with 1,800 pulses per session (approximately 9 min duration), and rMT determined at the beginning of each treatment week; whereas the TTT trial, as described elsewhere [13], employed an accelerated protocol with 3 sessions per day spaced 30 min apart over 15 weekdays (45 total sessions), with 1,200 pulses per session (approximately 6 min duration).

### Protocol comparison

To enable direct comparison of early response prediction across protocols, we matched assessment timepoints based on three complementary parameters. First, calendar time matching was performed at the end of weeks 1 and 2: the once-daily protocol week 1 (5 sessions) was compared with the accelerated protocol week 1 (15 sessions), and the once-daily protocol week 2 (10 sessions) was compared with the accelerated protocol week 2 (30 sessions). Second, pulse-based matching was implemented at 18,000 cumulative pulses, comparing the once-daily protocol week 2 (10 sessions) with the accelerated protocol week 1 (15 sessions). Third, session-based matching was implemented at 15 sessions, comparing the accelerated protocol week 1 (15 sessions) with the once-daily protocol week 3 (15 sessions), guided by recent evidence suggesting that number of sessions may be more relevant for treatment outcome than number of pulses [10]. We note that for the once-daily protocol, 15 sessions corresponds to week 3 of a 4-week treatment course (75% of treatment), which limits the interpretation of this timepoint as “early” prediction.

### Clinical assessments and outcomes

In both trials, depression severity was assessed using the 17-item Hamilton Depression Rating Scale (HDRS-17) [16], administered by trained raters at baseline and weekly intervals through the end of the acute treatment phase, with a final assessment 2 weeks post-treatment (once-daily protocol: week 6; accelerated protocol: week 5). Safety and tolerability were evaluated weekly using a standardized adverse events questionnaire.

### Early response and non-response prediction

The primary aim of this pooled analysis was to examine whether early symptom improvement could predict ultimate treatment outcome, and whether prediction patterns were consistent across accelerated and once-daily protocols. Early improvement was defined as ≥ 20% reduction in HDRS-17 from baseline, consistent with the threshold most commonly employed in the rTMS prediction literature [8, 9]. This threshold was used to classify participants as early improvers (≥ 20% reduction) versus early non-improvers (< 20% reduction). As a sensitivity analysis, we additionally examined ≥ 30% (response prediction) and < 10% (non-response prediction) thresholds (Supplementary Table S3).

### Statistical analysis

#### Primary efficacy analysis

Analyses followed a modified intention-to-treat approach including all participants who completed baseline and endpoint assessments. For the accelerated protocol, we analyzed only the active treatment arm (*n* = 50). Missing data during the treatment course were handled using Last Observation Carried Forward (LOCF) methodology. Response and remission rates were calculated with 95% confidence intervals using the Wilson score method. As a sensitivity analysis, we repeated all prediction analyses using only observed (non-imputed) data (Supplementary Tables S4 and S5).

Weekly symptom trajectories were analyzed using linear mixed-effects models with time as a fixed effect and random intercepts for participants. Post-hoc pairwise comparisons were conducted using estimated marginal means with false discovery rate correction for multiple comparisons.

#### Early response prediction analysis

For each protocol matching comparison, we calculated standard prediction metrics including sensitivity, specificity, positive predictive value (PPV), negative predictive value (NPV), and area under the receiver operating characteristic curve (AUC). The association between early and ultimate response was quantified using chi-square tests. To formally test whether the predictive relationship between early improvement and endpoint response differed between protocols, we fitted logistic regression models with endpoint response as the dependent variable. Model 1 included standardized early percent improvement, trial (TTT vs. BRAEN-MAP), and standardized baseline HDRS-17 severity as main effects. Model 2 added an early improvement × trial interaction term. A non-significant interaction indicates that the predictive relationship is equivalent across protocols. Models were compared using likelihood ratio tests (Supplementary Table S2).

### Comparison of predictive performance across protocols

To formally test whether positive predictive values (PPV) differed between protocols at each comparison timepoint, we employed two complementary approaches. First, we used bootstrap resampling (10,000 iterations) to estimate the sampling distribution of the PPV difference between trials, computing 95% percentile confidence intervals and two-sided p-values. Second, as a sensitivity analysis, we performed two-sample z-tests for proportions using pooled variance estimation. Individual PPV estimates and their 95% confidence intervals were calculated using the Wilson score method, which provides more accurate coverage than asymptotic methods for small sample sizes. These comparisons were conducted at each of the four matching timepoints: pulse-matched (18,000 pulses), time-matched Week 1, time-matched Week 2, and session-matched (15 sessions).

### Trajectory analysis by early response groups

To examine longitudinal symptom patterns, we classified participants into two groups based on early improvement at each matching timepoint: early improvers (≥ 20% HDRS-17 reduction) and early non-improvers (< 20% reduction). Symptom trajectories from baseline through endpoint were modeled using linear mixed-effects models with group (binary), time (continuous), and their interaction as fixed effects, and random intercepts for participants. A significant group × time interaction indicates that trajectory slopes differ between early improvers and non-improvers. LOCF was applied to ensure all participants contributed to trajectory models. Trajectory analyses were performed for all matching strategies.

All analyses were conducted using Python version 3.12 with the following packages: statsmodels for logistic regression and linear mixed-effects models; pandas for data manipulation; numpy for numerical operations; matplotlib for visualization; and scipy.stats for statistical computations including Wilson score confidence intervals.

## Results

### Sample characteristics

The BRAEN-MAP trial enrolled 65 participants, of whom 57 (87.7%) completed the full treatment protocol and endpoint assessment at week 6. Eight participants (12.3%) discontinued treatment: two due to adverse events (headache), three due to medical or family circumstances, and three were lost to follow-up.

The final analytic sample comprised 115 participants across both trials (BRAEN-MAP: *n* = 65; TTT active arm: *n* = 50) (Table 1). The two samples were comparable in age (*p* = 0.23) and sex distribution (*p* = 1.00). Reflecting the different inclusion criteria, the samples differed significantly in baseline HDRS-17 severity (BRAEN-MAP: 18.40 vs. TTT: 20.64; *p* < 0.001), number of failed antidepressant trials in the current episode (BRAEN-MAP: 2.0 vs. TTT: 3.6; *p* < 0.001), and proportion of participants on current antidepressant medication (BRAEN-MAP: 64.6% vs. TTT: 90.0%; *p* = 0.003).

**Table 1:** Sample Characteristics and Primary Outcomes

Variable	TTT Trial (*N* = 50)	BRAEN-MAP (*N* = 65)	*p*-value
Sample Size	50	65	-
Demographics			
Age, mean (SD)	43.5 (8.2)	41.2 (11.2)	0.226
Male, n (%)	8 (16.0%)	11 (16.9%)	1.000
Baseline Clinical			
HDRS-17, mean (SD)	20.64 (2.32)	18.40 (2.98)	< 0.001
Treatment resistance			
Failed AD trials (current episode), mean (SD)	3.6 (1.2)	2.0 (2.4)	< 0.001
Any current antidepressant	40 (90%)	42 (64.6%)	0.003
Treatment Parameters			
Sessions completed	45	20	-
Treatment duration	3 weeks	4 weeks	-
Target location	L-DLPFC	L-DLPFC	-
Positioning method	Beam F3	Beam F3	-
Stimulation intensity	100% rMT	100% rMT	-
Pulses per session	1200	1800	-
Pulses at the end of week 1	18,000	9000	-
Pulses at the end of week 2	36,000	18,000	-
Pulses at trial endpoint	54,000	36,000	
Primary Outcomes			
Endpoint assessment	Week 5 (2 weeks post-Tx)	Week 6 (2 weeks post-Tx)	-
HDRS-17 at endpoint	10.30 (5.49)	9.62 (5.99)	0.750
Change from baseline	10.34 (4.80)	8.78 (5.98)	0.120
Percent reduction	50.1%	47.7%	0.440
Effect size (Cohen’s d)	2,156	1,469	-
Response, n (%)	27/50 (54.0%)	36/65 (55.4%)	0.950
[95% CI]	[40.4–67.0]	[43.3–66.8]	
Remission, n (%)	17/50 (34.0%)	30/65 (46.2%)	0.200
[95% CI]	[22.4–47.8]	[34.6–58.1]	

*CI*: Confidence interval; *HDRS-17:* Hamilton Depression Rating Scale, 17-item version; *L-DLPFC :* Left dorsolateral prefrontal cortex; *rMT:*= resting motor threshold; *AD:* Antidepressant.

Demographic, clinical, treatment resistance, and outcome characteristics for both trials. TTT trial employed an accelerated protocol (3 sessions/day, 45 total sessions over 3 weeks) while BRAEN-MAP used a standard once-daily protocol (20 sessions over 4 weeks)

P-values reflect comparisons between trials using independent t-tests for continuous variables and Fisher's exact test or chi-square tests for categorical variables. Response defined as ≥50% reduction in HDRS-17 from baseline; remission defined as HDRS-17 ≤7 at endpoint

### Primary efficacy outcomes

Both trials demonstrated significant antidepressant effects. In BRAEN-MAP, HDRS-17 scores decreased from 18.40 (SD = 2.98) to 9.62 (SD = 5.99) at week 6, with 55.4% response rate (36/65) and 46.2% remission rate. In TTT, scores decreased from 20.64 (SD = 2.32) to 10.30 (SD = 5.49) at week 5, with 54.0% response rate (27/50) and 34.0% remission rate. There were no significant differences between trials in either response rates (*p* = 0.95) or endpoint severity (*p* = 0.75).

### Early response prediction

Early symptom improvement significantly predicted endpoint response across both protocols. Table 2 summarizes predictive performance at each assessment timepoint.

### Response prediction

For response prediction (PPV), values ranged from 66.7% to 75.0% across all protocol-timepoint combinations (Table 2). At the pulse-matched comparison (18,000 cumulative pulses), PPV was 66.7% (95% CI: 49.6–80.2%) in TTT (Week 1) versus 68.9% (95% CI: 54.3–80.5%) in BRAEN-MAP (Week 2), a non-significant difference of − 2.2% points (bootstrap *p* = 0.84). Time-matched comparisons similarly showed no significant differences: at Week 1, PPV was 66.7% in TTT versus 75.0% in BRAEN-MAP (difference: −8.3 pp, *p* = 0.45); at Week 2, PPV was 67.6% in TTT versus 68.9% in BRAEN-MAP (difference: −1.2 pp, *p* = 0.91). The session-matched comparison (15 sessions) yielded PPV of 66.7% in TTT versus 68.8% in BRAEN-MAP (difference: −2.1 pp, *p* = 0.86). No significant differences between protocols were observed at any matching strategy (all bootstrap *p* > 0.43).

### Non-response prediction

For non-response prediction (NPV), values ranged from 69.0% to 82.4%. In the pulse-matched comparison, NPV was 70.6% (95% CI: 46.9–86.7%) in TTT versus 75.0% (95% CI: 53.1–88.8%) in BRAEN-MAP (bootstrap *p* = 0.82). The session-matched comparison showed NPV of 70.6% in TTT versus 82.4% (95% CI: 59.0–93.8%) in BRAEN-MAP (difference: −11.8 pp, *p* = 0.43). The wide confidence intervals reflect the modest number of early non-improvers at some timepoints. NPV did not differ significantly between protocols at any matching strategy. Full diagnostic metrics including sensitivity, specificity, and AUC are presented in Supplementary Table S1.

### Comparison of matching strategies

Predictive performance was consistent across matching strategies. PPV and NPV values at the pulse-matched timepoint were comparable to both time-matched and session-matched comparisons, suggesting that the predictive relationship between early improvement and endpoint response is robust regardless of how equivalent dose is operationalized. This was confirmed by logistic regression equivalence testing: the early improvement × trial interaction was not significant at any matching strategy (all likelihood ratio test *p* > 0.60), indicating that the continuous relationship between early improvement and endpoint response did not differ between protocols after adjusting for baseline severity (Supplementary Table S2).

**Table 2:** Statistical Comparison of PPV Between Protocols

Prediction	Comparison	TTT (95% CI)	TTT *n*	BRAEN-MAP (95% CI)	BRAEN *n*	Difference (pp)	Diff 95% CI	*p*-value
Response (PPV)	Pulse-matched (18,000 pulses)	66.7% (49.6–80.2)	33	68.9% (54.3–80.5)	45	−2.2	−23.2 to 18.8	0.84
Response (PPV)	Time-matched Week 1	66.7% (49.6–80.2)	33	75.0% (58.9–86.2)	36	−8.3	−29.9 to 13.3	0.45
Response (PPV)	Time-matched Week 2	67.6% (50.8–80.9)	34	68.9% (54.3–80.5)	45	−1.2	−22.9 to 19.9	0.91
Response (PPV)	Session-matched (15 sessions)	66.7% (49.6–80.2)	33	68.8% (54.7–80.1)	48	−2.1	−23.4 to 18.7	0.86
Non-Response (NPV)	Pulse-matched (18,000 pulses)	70.6% (46.9–86.7)	17	75.0% (53.1–88.8)	20	−4.4	−33.9 to 25.0	0.82
Non-Response (NPV)	Time-matched Week 1	70.6% (46.9–86.7)	17	69.0% (50.8–82.7)	29	+ 1.6	−27.0 to 28.9	0.90
Non-Response (NPV)	Time-matched Week 2	75.0% (50.5–89.8)	16	75.0% (53.1–88.8)	20	0.0	−28.9 to 28.8	1.00
Non-Response (NPV)	Session-matched (15 sessions)	70.6% (46.9–86.7)	17	82.4% (59.0–93.8)	17	−11.8	−40.2 to 16.7	0.43

Positive predictive values (PPV, for response prediction) and negative predictive values (NPV, for non-response prediction) using ≥20% early HDRS-17 improvement at four matching timepoints: pulse-matched (18,000 cumulative pulses: TTT Week 1 vs. BRAEN-MAP Week 2), time-matched Week 1 (TTT Week 1 vs. BRAEN-MAP Week 1), time-matched Week 2 (TTT Week 2 vs. BRAEN-MAP Week 2), and session-matched (15 sessions: TTT Week 1 vs. BRAEN-MAP Week 3). PPV estimates and 95% confidence intervals calculated using the Wilson score method. Between-protocol differences tested using bootstrap resampling (10,000 iterations). No significant differences were observed between protocols at any timepoint. TTT = accelerated theta-burst stimulation trial; BRAEN-MAP = standard once-daily theta-burst stimulation trial.

### Trajectory analysis

**Fig. 1:**
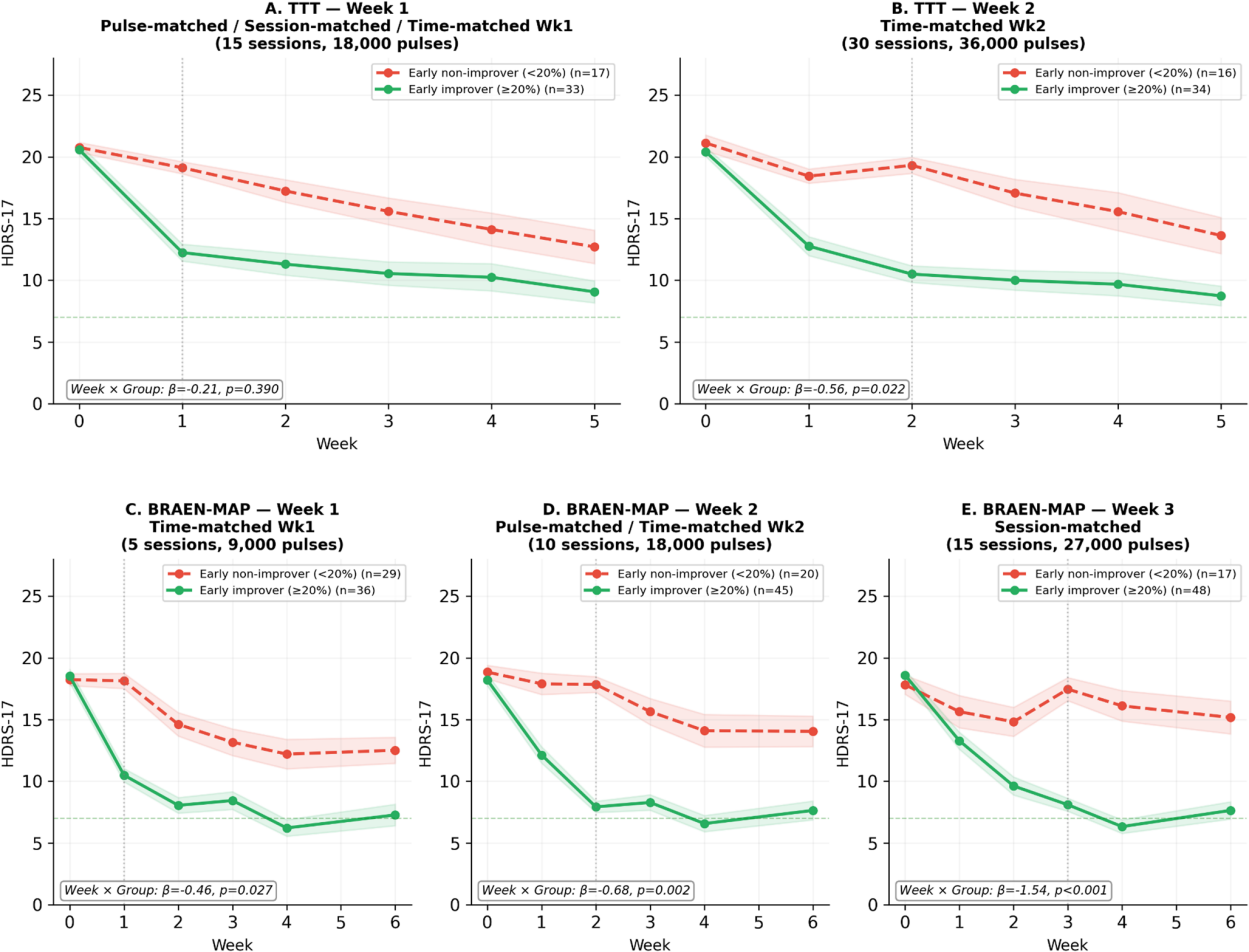
Symptom Trajectories by Early Improvement Group Across Matching Strategies

Symptom trajectories were examined across all unique classification timepoints (Fig. 1). In each analysis, participants were independently classified as early improvers (≥ 20% HDRS-17 reduction) or early non-improvers (< 20% reduction) at the respective assessment timepoint, and trajectories were modeled from baseline through endpoint.

In TTT, when participants were classified at Week 1 (pulse-matched and session-matched; 15 sessions/18,000 pulses), the week × group interaction was not significant (β=−0.21, *p* = 0.39), indicating that early improvers and non-improvers declined at similar rates through endpoint. When classifying participants at Week 2 (time-matched; 30 sessions), the interaction was significant (β=−0.57, *p* = 0.022), indicating diverging trajectories, with early improvers showing a steeper decline than non-improvers.

In BRAEN-MAP, the week × group interaction was significant at all three classification timepoints: Week 1 (time-matched Week 1; 5 sessions: β=−0.46, *p* = 0.027), Week 2 (pulse-matched and time-matched Week 2; 10 sessions: β=−0.68, *p* = 0.002), and Week 3 (session-matched; 15 sessions: β=−1.54, *p* < 0.001), with the magnitude of divergence increasing at later assessment points.

Mean HDRS-17 scores from baseline through endpoint for participants classified as early improvers (≥ 20% HDRS-17 reduction; solid lines) versus early non-improvers (< 20% reduction; dashed lines). Shaded areas represent standard error of the mean. Vertical dotted lines indicate the assessment timepoint at which participants were classified. The horizontal dashed line indicates the remission threshold (HDRS-17 ≤ 7). Top row: TTT (accelerated protocol). Panel A: classified at Week 1 (15 sessions/18,000 pulses; serves pulse-matched, session-matched, and time-matched Week 1 comparisons). Panel B: classified at Week 2 (30 sessions/36,000 pulses; time-matched Week 2). Bottom row: BRAEN-MAP (once-daily protocol). Panel C: classified at Week 1 (5 sessions/9,000 pulses; time-matched Week 1). Panel D: classified at Week 2 (10 sessions/18,000 pulses; serves pulse-matched and time-matched Week 2 comparisons). Panel E: classified at Week 3 (15 sessions/27,000 pulses; session-matched). Week × group interaction statistics are annotated on each panel; a significant interaction indicates diverging trajectory slopes between groups. BRAEN-MAP = Brazil-England Study on Mechanisms and Response Predictors of iTBS Treatment; HDRS-17 = Hamilton Depression Rating Scale, 17-item version; TTT = Triple TBS Thirty-minute Interval trial.

## Discussion

This study analyzed early response to iTBS treatment as a predictor of treatment outcome in two clinical trials employing different delivery schedules: a standard once-daily protocol (BRAEN-MAP) and an accelerated protocol with three daily sessions (TTT). Using a ≥ 20% early improvement threshold, we compared predictive performance across pulse-matched (18,000 pulses), time-matched (Weeks 1 and 2), and session-matched (15 sessions) assessment points.

For BRAEN-MAP, this represents the first report of clinical outcomes from this open-label trial. For TTT, this study extends prior analyses by examining prediction consistency across protocols. BRAEN-MAP trial demonstrated a response rate of 55.4% and remission rate of 46.2%, which did not differ significantly from the TTT trial (54.0% and 34.0%, respectively). Endpoint response rates were equivalent despite TTT delivering more than twice the number of sessions (45 vs. 20). While this observation should be interpreted cautiously given the non-randomized comparison and differing baseline characteristics, it is noteworthy in light of Oostra et al. (2025) [10], who found in a meta-regression of 87 RCTs that the number of sessions significantly predicted treatment outcome for HF-rTMS and LF-rTMS. However, that meta-analysis explicitly excluded iTBS and cTBS protocols, so it remains unclear whether the same session-dependent dose-response relationship applies to theta-burst stimulation. The comparable outcomes observed here despite a large session count difference may reflect distinct dose-response characteristics of iTBS relative to conventional rTMS protocols.

Our primary finding is that the predictive relationship between early improvement and endpoint response did not differ significantly between protocols at any matching strategy. PPV for response prediction ranged from 66.7% to 75.0% across all protocol-timepoint combinations, with no significant differences between protocols (all bootstrap *p* > 0.43). NPV for non-response prediction ranged from 69.0% to 82.4%, also without significant between-protocol differences (all bootstrap *p* > 0.43). The consistency of predictive performance across all matching strategies suggests that the early predictive signal is robust to how “equivalent dose” is operationalized. If, as suggested by Oostra et al. [10], sessions represent the more clinically meaningful dosing unit, then the session-matched comparison may offer the most appropriate framework for evaluating prediction equivalence. Yet PPV and NPV did not differ significantly under any matching strategy, including session-matched, suggesting that the early predictive signal is independent of the specific dosing parameter used for assessment timing. Whether this consistency extends to iTBS specifically, given its exclusion from the Oostra et al. analysis, warrants further investigation with larger samples.

These findings also align with converging evidence from larger studies. Sackeim et al. (2024) [9], analyzing over 7,000 patients from a clinical registry, demonstrated that early symptomatic improvement is strongly predictive of eventual response. Mirman et al. (2022) [8], in a naturalistic study of 897 patients receiving at least 30 sessions of rTMS, found PPV values exceeding 70% for response on the PHQ and HDRS at sessions 5 and 10. Our observation of 66.7–75.0% PPV across both protocols is consistent with these findings and extends them to iTBS-specific contexts, including accelerated delivery schedules not previously examined.

The interpretation of early non-improvement as a predictor of non-response warrants caution. Although NPV ranged from 68.9% to 82.4% across timepoints, the wide confidence intervals reflect modest sample sizes and preclude conclusions about potential differences in non-response prediction between protocols. More broadly, recent literature has questioned the reliability of non-response prediction in rTMS. Sackeim et al. (2024) [9] demonstrated that positive predictive values for nonresponse detection were only 51–63% after 10 sessions when patients completed a full 36-session course, meaning that a substantial proportion of patients classified as likely nonresponders based on early lack of improvement ultimately responded by treatment end. Beck et al. (2020) [17] further showed that prediction accuracy decreased by approximately 20% points when outcomes were assessed after a full 36-session course compared to 20 sessions, providing evidence for a clinically meaningful late-responder population. These findings are consistent with Oostra et al.‘s [10] observation that the number of sessions significantly predicts treatment outcome: if additional sessions allow more patients to cross the response threshold, then early non-improvement becomes an increasingly unreliable predictor as treatment courses are extended. As BRAEN-MAP delivered only 20 sessions, non-response prediction may have been somewhat inflated, although both protocols yielded comparable NPV estimates despite differing in total session count (20 vs. 45).

Regarding the clinical application of early response prediction, current evidence, including our findings, does not support using early non-improvement as a basis for treatment discontinuation. Across studies, predicting response has proven considerably more accurate than predicting nonresponse [8, 9]. Rather than supporting binary stop/continue decisions, early prediction may be most useful within an adaptive, measurement-based care framework. Lee et al. (2020) [12] demonstrated that patients with < 20% improvement after 10 sessions who continued unchanged high-frequency left-sided rTMS showed limited benefit (12% response rate), whereas those whose protocol was augmented with intermittent theta-burst priming achieved substantially greater improvement (41% response rate; d = 0.80), with approximately 10 post-switch sessions required to evaluate the new approach. This suggests that early non-improvement should not prompt treatment cessation, but rather systematic evaluation of whether protocol modification may improve the likelihood of response. Our finding that early prediction operates similarly across standard and accelerated protocols supports applying this adaptive approach regardless of delivery schedule, identifying patients who may benefit from treatment intensification while preserving the therapeutic opportunity for late responders.

Notably, the sample characterization revealed significant baseline differences between trials in depression severity (*p* < 0.001), number of failed antidepressant trials (*p* < 0.001), and antidepressant use (*p* = 0.003), reflecting the different inclusion criteria. That early prediction accuracy remained equivalent despite these clinical differences suggests that the predictive relationship between early improvement and endpoint response is robust across varying levels of treatment resistance and baseline severity, which strengthens the potential generalizability of this clinical tool. Moreover, both trials were conducted at the same site with access to the same patient population and evaluated by the same clinical team, reducing the influence of unmeasured demographic or rater-related confounders on between-protocol comparisons.

This study has important strengths. The comparison of two independent trials with different treatment intensities—standard once-daily versus accelerated three-times-daily protocols—allowed examination of whether early prediction generalizes across iTBS delivery schedules. Both trials were conducted by the same research group, using similar methodology and overlapping team members, which further strengthens the comparativeness. The inclusion of four matching strategies (pulse-matched, two time-matched, and session-matched) provides a comprehensive evaluation of prediction consistency across different operationalizations of equivalent dose.

Several limitations are also important. BRAEN-MAP was an open-label trial without sham control, and expectancy effects may have influenced both symptom improvement and early prediction patterns; although TTT included sham-controlled data, our analysis focused on the active arm to maximize comparability. Furthermore, comparing an open-label trial with the active arm of a sham-controlled RCT introduces a potential asymmetry: patients in the RCT who are aware of possible sham allocation may exhibit lower treatment expectations, a phenomenon described as the “nocebo effect,” which could attenuate symptom improvement in the active arm [18]. This expectancy asymmetry could influence both response rates and the calibration of early prediction thresholds. The inclusion criteria differed between trials: BRAEN-MAP enrolled patients with MDD using a lower baseline severity threshold (HDRS-17 ≥ 14), whereas the randomized TTT trial was restricted to treatment-resistant depression with higher baseline severity (HDRS-17 ≥ 17), and the samples differed significantly on baseline severity, treatment resistance, and medication use. The protocols differed substantially in total session count (45 vs. 20), and as recent evidence suggests sessions may be more predictive of outcome than pulses [10], this difference may influence endpoint outcomes and the interpretation of prediction across protocols. The trials had different endpoint assessments (Week 5 for TTT, Week 6 for BRAEN-MAP), though both represented two weeks post-treatment completion. Sample sizes were modest (*N* = 115 combined), and particularly the number of early non-improvers at some timepoints was small, limiting statistical power for NPV comparisons. The proportion of men was low in both samples (TTT: 16.0%; BRAEN-MAP: 16.9%), which limits the generalizability of our findings to male patients given that biomarker outcomes may be sex-specific [7]. The proposed thresholds were derived retrospectively and require prospective validation before clinical implementation. Both trials used the same depression measure (HDRS-17), stimulation target (left DLPFC via Beam F3), and intensity (100% rMT), which may limit generalizability to protocols using different parameters. Our analysis cannot distinguish whether minimal early responders are true non-responders who will not benefit regardless of treatment duration, or slower responders who might eventually improve with continued stimulation, a distinction with important clinical implications. Most importantly, this was not a randomized comparison, but rather a post-hoc analysis of two trials, therefore confirmatory randomized clinical trials directly comparing accelerated and standard protocols with pre-specified early prediction endpoints are necessary to validate these findings and establish clinical decision frameworks.

## Conclusion

Early symptom improvement consistently predicted iTBS response across accelerated and standard protocols, with positive predictive values of 66.7–75.0% regardless of whether assessments were matched by cumulative pulse exposure, calendar time, or number of sessions. Patients achieving ≥ 20% early improvement showed significantly steeper symptom trajectories through endpoint. Non-response prediction showed limited precision, with wide confidence intervals reflecting modest sample sizes, and evidence for late responders in extended treatment courses suggests caution in using minimal early improvement to guide treatment decisions. Rather than justifying treatment discontinuation, early response assessment may be most useful within adaptive, measurement-based care frameworks that guide protocol modification for early non-improvers. These preliminary findings, derived from post-hoc analysis of two independent trials with different populations, require validation in prospective randomized studies directly comparing protocols with pre-specified early prediction endpoints.

## Supplementary Information

Below is the link to the electronic supplementary material.

Supplementary Material 1

## Data Availability

The datasets generated and analyzed during the current study are available from the corresponding author upon reasonable request.
