# Supplementary material for: Dose or time? Comparing early prediction of intermittent theta-burst stimulation response across accelerated and daily protocols for depression
Source: Eur Arch Psychiatry Clin Neurosci. 2026 Jun 1;276(5):2537–46. doi: 10.1007/s00406-026-02266-2 (PMC13481425; doi:10.1007/s00406-026-02266-2)
Supplement: Supplementary file 1 [file 406_2026_2266_MOESM1_ESM.docx]

**Supplementary Materials**

*Dose or time? Comparing early prediction of intermittent theta-burst stimulation response across accelerated and daily protocols for depression*

## Table S1: Full Diagnostic Metrics at ≥20% Early Improvement Threshold

*Complete prediction metrics for the primary ≥20% early improvement threshold across all four matching strategies. PPV = positive predictive value (proportion of early improvers who achieve endpoint response); NPV = negative predictive value (proportion of early non-improvers who do not achieve endpoint response). Sensitivity = proportion of endpoint responders correctly classified as early improvers; Specificity = proportion of endpoint non-responders correctly classified as early non-improvers. AUC = area under the receiver operating characteristic curve. OR = odds ratio with 95% confidence interval. CI = confidence interval calculated using the Wilson score method. N = total sample; TP = true positives; FP = false positives; FN = false negatives; TN = true negatives.*

| **Comparison** | **Trial** | **N** | **Sens (%)** | **Spec (%)** | **PPV (95% CI)** | **NPV (95% CI)** | **AUC** | **OR [95% CI]** |
| --- | --- | --- | --- | --- | --- | --- | --- | --- |
| Pulse-matched (18,000 pulses) | TTT | 50 | 81.5 | 52.2 | 66.7 (49.6-80.2) | 70.6 (46.9-86.7) | 0.668 | 4.80 (1.35-17.09) |
| Pulse-matched (18,000 pulses) | BRAEN-MAP | 65 | 86.1 | 51.7 | 68.9 (54.3-80.5) | 75.0 (53.1-88.8) | 0.689 | 6.64 (2.02-21.90) |
| Time-matched Week 1 | TTT | 50 | 81.5 | 52.2 | 66.7 (49.6-80.2) | 70.6 (46.9-86.7) | 0.668 | 4.80 (1.35-17.09) |
| Time-matched Week 1 | BRAEN-MAP | 65 | 75.0 | 69.0 | 75.0 (58.9-86.2) | 69.0 (50.8-82.7) | 0.720 | 6.67 (2.24-19.83) |
| Time-matched Week 2 | TTT | 50 | 85.2 | 52.2 | 67.6 (50.8-80.9) | 75.0 (50.5-89.8) | 0.687 | 6.27 (1.64-23.97) |
| Time-matched Week 2 | BRAEN-MAP | 65 | 86.1 | 51.7 | 68.9 (54.3-80.5) | 75.0 (53.1-88.8) | 0.689 | 6.64 (2.02-21.90) |
| Session-matched (15 sessions) | TTT | 50 | 81.5 | 52.2 | 66.7 (49.6-80.2) | 70.6 (46.9-86.7) | 0.668 | 4.80 (1.35-17.09) |
| Session-matched (15 sessions) | BRAEN-MAP | 65 | 91.7 | 48.3 | 68.8 (54.7-80.1) | 82.4 (59.0-93.8) | 0.700 | 10.27 (2.56-41.15) |

## Table S2: Protocol Equivalence Testing — Logistic Regression Models

| **Parameter** | **β** | **SE** | **z** | **p-value** | **OR** | **OR 95% CI** | **Sig** |
| --- | --- | --- | --- | --- | --- | --- | --- |
| **Pulse-matched (18,000 pulses)** |  |  |  |  |  |  |  |
| **Model 1** |  |  |  |  |  |  |  |
| Intercept | -0.050 | 0.294 | -0.17 | 0.865 | 0.95 | [0.53, 1.69] |  |
| pct_improvement_std | 0.994 | 0.244 | 4.08 | <0.001 | 2.70 | [1.68, 4.36] | *** |
| trial_ttt | 0.665 | 0.472 | 1.41 | 0.159 | 1.94 | [0.77, 4.90] |  |
| baseline_std | -0.384 | 0.235 | -1.63 | 0.103 | 0.68 | [0.43, 1.08] |  |
| **Model 2** |  |  |  |  |  |  |  |
| Intercept | -0.040 | 0.291 | -0.14 | 0.890 | 0.96 | [0.54, 1.70] |  |
| pct_improvement_std | 0.931 | 0.286 | 3.25 | 0.001 | 2.54 | [1.45, 4.45] | ** |
| trial_ttt | 0.705 | 0.486 | 1.45 | 0.147 | 2.02 | [0.78, 5.24] |  |
| pct_improvement_std:trial_ttt | 0.213 | 0.539 | 0.40 | 0.692 | 1.24 | [0.43, 3.56] |  |
| baseline_std | -0.380 | 0.235 | -1.62 | 0.105 | 0.68 | [0.43, 1.08] |  |
| **LR Test (Interaction)** |  |  |  |  |  |  |  |
| χ² statistic | 0.160 | - | - | 0.689 |  |  | ns |
| **Time-matched Week 1** |  |  |  |  |  |  |  |
| **Model 1** |  |  |  |  |  |  |  |
| Intercept | 0.220 | 0.284 | 0.77 | 0.438 | 1.25 | [0.71, 2.18] |  |
| pct_improvement_std | 0.874 | 0.233 | 3.75 | <0.001 | 2.40 | [1.52, 3.78] | *** |
| trial_ttt | 0.054 | 0.444 | 0.12 | 0.903 | 1.06 | [0.44, 2.52] |  |
| baseline_std | -0.402 | 0.231 | -1.74 | 0.082 | 0.67 | [0.43, 1.05] |  |
| **Model 2** |  |  |  |  |  |  |  |
| Intercept | 0.207 | 0.282 | 0.73 | 0.463 | 1.23 | [0.71, 2.14] |  |
| pct_improvement_std | 0.789 | 0.280 | 2.82 | 0.005 | 2.20 | [1.27, 3.81] | ** |
| trial_ttt | 0.062 | 0.446 | 0.14 | 0.889 | 1.06 | [0.44, 2.55] |  |
| pct_improvement_std:trial_ttt | 0.259 | 0.503 | 0.52 | 0.606 | 1.30 | [0.48, 3.47] |  |
| baseline_std | -0.394 | 0.230 | -1.71 | 0.087 | 0.67 | [0.43, 1.06] |  |
| **LR Test (Interaction)** |  |  |  |  |  |  |  |
| χ² statistic | 0.272 | - | - | 0.602 |  |  | ns |
| **Time-matched Week 2** |  |  |  |  |  |  |  |
| **Model 1** |  |  |  |  |  |  |  |
| Intercept | 0.064 | 0.292 | 0.22 | 0.826 | 1.07 | [0.60, 1.89] |  |
| pct_improvement_std | 1.033 | 0.239 | 4.32 | <0.001 | 2.81 | [1.76, 4.49] | *** |
| trial_ttt | 0.402 | 0.464 | 0.87 | 0.386 | 1.50 | [0.60, 3.71] |  |
| baseline_std | -0.356 | 0.240 | -1.48 | 0.138 | 0.70 | [0.44, 1.12] |  |
| **Model 2** |  |  |  |  |  |  |  |
| Intercept | 0.069 | 0.288 | 0.24 | 0.811 | 1.07 | [0.61, 1.88] |  |
| pct_improvement_std | 0.954 | 0.294 | 3.25 | 0.001 | 2.60 | [1.46, 4.62] | ** |
| trial_ttt | 0.422 | 0.468 | 0.90 | 0.368 | 1.52 | [0.61, 3.82] |  |
| pct_improvement_std:trial_ttt | 0.220 | 0.504 | 0.44 | 0.662 | 1.25 | [0.46, 3.34] |  |
| baseline_std | -0.348 | 0.239 | -1.45 | 0.146 | 0.71 | [0.44, 1.13] |  |
| **LR Test (Interaction)** |  |  |  |  |  |  |  |
| χ² statistic | 0.194 | - | - | 0.660 |  |  | ns |
| **Session-matched (15 sessions)** |  |  |  |  |  |  |  |
| **Model 1** |  |  |  |  |  |  |  |
| Intercept | -0.162 | 0.312 | -0.52 | 0.602 | 0.85 | [0.46, 1.57] |  |
| pct_improvement_std | 1.295 | 0.281 | 4.62 | <0.001 | 3.65 | [2.11, 6.33] | *** |
| trial_ttt | 0.965 | 0.511 | 1.89 | 0.059 | 2.62 | [0.96, 7.14] |  |
| baseline_std | -0.472 | 0.262 | -1.80 | 0.071 | 0.62 | [0.37, 1.04] |  |
| **Model 2** |  |  |  |  |  |  |  |
| Intercept | -0.173 | 0.318 | -0.54 | 0.587 | 0.84 | [0.45, 1.57] |  |
| pct_improvement_std | 1.341 | 0.344 | 3.89 | <0.001 | 3.82 | [1.95, 7.51] | *** |
| trial_ttt | 0.941 | 0.519 | 1.81 | 0.070 | 2.56 | [0.93, 7.08] |  |
| pct_improvement_std:trial_ttt | -0.139 | 0.587 | -0.24 | 0.812 | 0.87 | [0.28, 2.75] |  |
| baseline_std | -0.477 | 0.264 | -1.81 | 0.071 | 0.62 | [0.37, 1.04] |  |
| **LR Test (Interaction)** |  |  |  |  |  |  |  |
| χ² statistic | 0.056 | - | - | 0.813 |  |  | ns |

*Logistic regression models testing whether the predictive relationship between early improvement and endpoint response differs between protocols. For each matching strategy, Model 1 includes main effects of standardized percent improvement, trial (TTT=1), and standardized baseline severity. Model 2 adds the improvement × trial interaction. A non-significant interaction indicates equivalent predictive relationships across protocols. The likelihood ratio (LR) test formally compares model fit. Coefficients are on the log-odds scale; OR = exponentiated coefficient. pct_improvement_std = standardized percentage improvement from baseline at the assessment timepoint; trial_ttt = trial indicator (TTT=1, BRAEN-MAP=0); baseline_std = standardized baseline HDRS-17 score. * p<0.05, ** p<0.01, *** p<0.001, ns = not significant.*

## Table S3: Sensitivity Analysis — Prediction at Alternative Thresholds (≥30% / <10%)

| **Comparison** | **Prediction** | **TTT (95% CI)** | **BRAEN-MAP (95% CI)** | **Diff (pp)** | **Diff 95% CI** | **p** |
| --- | --- | --- | --- | --- | --- | --- |
| Pulse-matched (18,000 pulses) | Response (>=30%) | 77.3% (56.6-89.9) | 73.2% (58.1-84.3) | +4.1 | (-19.3 to 25.6) | 0.723 |
| Pulse-matched (18,000 pulses) | Non-Response (<10%) | 88.9% (56.5-98.0) | 66.7% (39.1-86.2) | +22.2 | (-13.3 to 57.1) | 0.240 |
| Time-matched Week 1 | Response (>=30%) | 77.3% (56.6-89.9) | 71.4% (52.9-84.7) | +5.8 | (-19.0 to 29.8) | 0.652 |
| Time-matched Week 1 | Non-Response (<10%) | 88.9% (56.5-98.0) | 71.4% (50.0-86.2) | +17.5 | (-14.3 to 44.7) | 0.262 |
| Time-matched Week 2 | Response (>=30%) | 71.0% (53.4-83.9) | 73.2% (58.1-84.3) | -2.2 | (-23.3 to 18.5) | 0.836 |
| Time-matched Week 2 | Non-Response (<10%) | 83.3% (43.6-97.0) | 66.7% (39.1-86.2) | +16.7 | (-30.5 to 56.2) | 0.455 |
| Session-matched (15 sessions) | Response (>=30%) | 77.3% (56.6-89.9) | 74.4% (59.8-85.1) | +2.9 | (-19.6 to 24.5) | 0.803 |
| Session-matched (15 sessions) | Non-Response (<10%) | 88.9% (56.5-98.0) | 83.3% (55.2-95.3) | +5.6 | (-25.7 to 36.4) | 0.789 |

*Prediction performance using alternative thresholds: ≥30% early HDRS-17 improvement for response prediction and <10% early improvement for non-response prediction. These thresholds were examined as sensitivity analyses to complement the primary ≥20% threshold analysis reported in Table 2 of the main manuscript. PPV for response prediction represents the proportion of patients with ≥30% early improvement who achieved endpoint response. PPV for non-response prediction represents the proportion of patients with <10% early improvement who did not achieve endpoint response. CI = confidence interval (Wilson score method). Bootstrap p-values from 10,000 resampling iterations. No significant differences between protocols were observed at any matching strategy.*

## Table S4: Sensitivity Analysis — Complete-Case Prediction (Observed Data Only)

*Prediction analysis repeated using only observed (non-imputed) data, excluding participants with missing endpoint assessments. This tests whether LOCF imputation materially influenced prediction estimates. PPV = positive predictive value (response prediction); NPV = negative predictive value (non-response prediction). Sample sizes are reduced relative to the primary analysis due to exclusion of participants requiring LOCF. Bootstrap p-values from 10,000 iterations. Results are consistent with the primary LOCF analysis, supporting the robustness of findings.*

| **Comparison** | **Metric** | **Trial** | **N** | **Value (95% CI)** | **Diff (pp)** | **Diff 95% CI** | **Bootstrap p** |
| --- | --- | --- | --- | --- | --- | --- | --- |
| Pulse-matched (18,000 pulses) | PPV (Response) | TTT | 47 | 66.7% (49.6-80.2) | -2.2 | (-23.2 to 18.6) | 0.840 |
| Pulse-matched (18,000 pulses) | PPV (Response) | BRAEN-MAP | 60 | 68.9% (54.3-80.5) | -2.2 | (-23.2 to 18.6) | 0.840 |
| Pulse-matched (18,000 pulses) | NPV (Non-Response) | TTT | 47 | 64.3% (38.8-83.7) | -2.4 | (-38.5 to 33.3) | 0.927 |
| Pulse-matched (18,000 pulses) | NPV (Non-Response) | BRAEN-MAP | 60 | 66.7% (41.7-84.8) | -2.4 | (-38.5 to 33.3) | 0.927 |
| Time-matched Week 1 | PPV (Response) | TTT | 47 | 66.7% (49.6-80.2) | -8.3 | (-29.9 to 13.2) | 0.457 |
| Time-matched Week 1 | PPV (Response) | BRAEN-MAP | 60 | 75.0% (58.9-86.2) | -8.3 | (-29.9 to 13.2) | 0.457 |
| Time-matched Week 1 | NPV (Non-Response) | TTT | 47 | 64.3% (38.8-83.7) | +1.8 | (-31.1 to 33.3) | 0.922 |
| Time-matched Week 1 | NPV (Non-Response) | BRAEN-MAP | 60 | 62.5% (42.7-78.8) | +1.8 | (-31.1 to 33.3) | 0.922 |
| Time-matched Week 2 | PPV (Response) | TTT | 46 | 66.7% (49.6-80.2) | -2.2 | (-23.3 to 19.2) | 0.856 |
| Time-matched Week 2 | PPV (Response) | BRAEN-MAP | 60 | 68.9% (54.3-80.5) | -2.2 | (-23.3 to 19.2) | 0.856 |
| Time-matched Week 2 | NPV (Non-Response) | TTT | 46 | 69.2% (42.4-87.3) | +2.6 | (-34.5 to 37.6) | 0.922 |
| Time-matched Week 2 | NPV (Non-Response) | BRAEN-MAP | 60 | 66.7% (41.7-84.8) | +2.6 | (-34.5 to 37.6) | 0.922 |
| Session-matched (15 sessions) | PPV (Response) | TTT | 47 | 66.7% (49.6-80.2) | -1.4 | (-22.2 to 19.4) | 0.916 |
| Session-matched (15 sessions) | PPV (Response) | BRAEN-MAP | 57 | 68.1% (53.8-79.6) | -1.4 | (-22.2 to 19.4) | 0.916 |
| Session-matched (15 sessions) | NPV (Non-Response) | TTT | 47 | 64.3% (38.8-83.7) | -5.7 | (-44.4 to 35.7) | 0.775 |
| Session-matched (15 sessions) | NPV (Non-Response) | BRAEN-MAP | 57 | 70.0% (39.7-89.2) | -5.7 | (-44.4 to 35.7) | 0.775 |

## Table S5: Sensitivity Analysis — Complete-Case Trajectory Models (Observed Data Only)

*Linear mixed-effects trajectory models using only observed (non-imputed) data, requiring at least 3 observed timepoints per participant. The week × improver interaction tests whether symptom trajectory slopes differ between early improvers (≥20% HDRS-17 reduction) and early non-improvers (<20% reduction). A significant interaction indicates diverging trajectories. Participants with fewer than 3 observed timepoints were excluded. Group Var = random intercept variance.*

| **Dataset** | **Parameter** | **Estimate** | **SE** | **z** | **p** |
| --- | --- | --- | --- | --- | --- |
| Pulse-matched: TTT Wk1 | Intercept | 20.653 | 1.124 | 18.38 | <0.001 *** |
| Pulse-matched: TTT Wk1 | week | -1.976 | 0.221 | -8.94 | <0.001 *** |
| Pulse-matched: TTT Wk1 | improver | -3.628 | 1.348 | -2.69 | 0.007 ** |
| Pulse-matched: TTT Wk1 | **week:improver** | 0.061 | 0.266 | 0.23 | 0.819 |
| Pulse-matched: TTT Wk1 | Group Var | 0.953 | 0.263 | 3.62 | <0.001 *** |
| Pulse-matched: BRAEN Wk2 | Intercept | 18.823 | 0.926 | 20.33 | <0.001 *** |
| Pulse-matched: BRAEN Wk2 | week | -1.363 | 0.234 | -5.83 | <0.001 *** |
| Pulse-matched: BRAEN Wk2 | improver | -4.441 | 1.068 | -4.16 | <0.001 *** |
| Pulse-matched: BRAEN Wk2 | **week:improver** | -0.229 | 0.268 | -0.85 | 0.393 |
| Pulse-matched: BRAEN Wk2 | Group Var | 0.271 | 0.090 | 3.02 | 0.003 ** |
| Time-matched Wk1: BRAEN Wk1 | Intercept | 17.593 | 0.763 | 23.05 | <0.001 *** |
| Time-matched Wk1: BRAEN Wk1 | week | -1.458 | 0.186 | -7.84 | <0.001 *** |
| Time-matched Wk1: BRAEN Wk1 | improver | -3.516 | 0.983 | -3.58 | <0.001 *** |
| Time-matched Wk1: BRAEN Wk1 | **week:improver** | -0.126 | 0.235 | -0.54 | 0.592 |
| Time-matched Wk1: BRAEN Wk1 | Group Var | 0.332 | 0.103 | 3.22 | 0.001 ** |
| Time-matched Wk2: TTT Wk2 | Intercept | 21.071 | 1.094 | 19.26 | <0.001 *** |
| Time-matched Wk2: TTT Wk2 | week | -1.743 | 0.230 | -7.57 | <0.001 *** |
| Time-matched Wk2: TTT Wk2 | improver | -4.099 | 1.292 | -3.17 | 0.002 ** |
| Time-matched Wk2: TTT Wk2 | **week:improver** | -0.268 | 0.272 | -0.98 | 0.326 |
| Time-matched Wk2: TTT Wk2 | Group Var | 0.782 | 0.222 | 3.52 | <0.001 *** |
| Time-matched Wk2: BRAEN Wk2 | Intercept | 18.823 | 0.926 | 20.33 | <0.001 *** |
| Time-matched Wk2: BRAEN Wk2 | week | -1.363 | 0.234 | -5.83 | <0.001 *** |
| Time-matched Wk2: BRAEN Wk2 | improver | -4.441 | 1.068 | -4.16 | <0.001 *** |
| Time-matched Wk2: BRAEN Wk2 | **week:improver** | -0.229 | 0.268 | -0.85 | 0.393 |
| Time-matched Wk2: BRAEN Wk2 | Group Var | 0.271 | 0.090 | 3.02 | 0.003 ** |
| Session-matched: TTT Wk1 | Intercept | 20.653 | 1.124 | 18.38 | <0.001 *** |
| Session-matched: TTT Wk1 | week | -1.976 | 0.221 | -8.94 | <0.001 *** |
| Session-matched: TTT Wk1 | improver | -3.628 | 1.348 | -2.69 | 0.007 ** |
| Session-matched: TTT Wk1 | **week:improver** | 0.061 | 0.266 | 0.23 | 0.819 |
| Session-matched: TTT Wk1 | Group Var | 0.953 | 0.263 | 3.62 | <0.001 *** |
| Session-matched: BRAEN Wk3 | Intercept | 15.874 | 1.117 | 14.21 | <0.001 *** |
| Session-matched: BRAEN Wk3 | week | -0.403 | 0.255 | -1.58 | 0.114 |
| Session-matched: BRAEN Wk3 | improver | -0.497 | 1.248 | -0.40 | 0.691 |
| Session-matched: BRAEN Wk3 | **week:improver** | -1.391 | 0.282 | -4.93 | <0.001 *** |
| Session-matched: BRAEN Wk3 | Group Var | 0.466 | 0.131 | 3.57 | <0.001 *** |

To assess the robustness of findings to the LOCF imputation strategy, all prediction and trajectory analyses were repeated using only observed (non-imputed) data. For prediction analyses (Table S4), sample sizes were reduced to N=46–47 (TTT) and N=57–60 (BRAEN-MAP) depending on timepoint and data availability. Results were consistent with the primary LOCF analysis: PPV for response prediction ranged from 66.7% to 75.0%, and no significant differences between protocols were observed at any matching strategy (all bootstrap p>0.45). NPV estimates were also comparable, ranging from 62.5% to 70.0%.

For trajectory analyses (Table S5), the requirement of at least 3 observed timepoints per participant reduced sample sizes modestly. The pattern of results was largely consistent with the primary analysis, though some interactions that were significant in the LOCF analysis did not reach significance in the complete-case analysis, likely reflecting reduced statistical power. Specifically, the TTT Week 2 time-matched trajectory interaction was not significant in the complete-case analysis (β=−0.27, p=0.326), compared to β=−0.57, p=0.022 in the LOCF analysis. The BRAEN-MAP Week 2 pulse-matched/time-matched interaction was also attenuated (β=−0.23, p=0.393 vs. β=−0.68, p=0.002 in LOCF). BRAEN-MAP session-matched (Week 3) trajectories remained strongly significant (β=−1.39, p<0.001). Overall, complete-case results support the robustness of the primary findings, with the attenuation of some trajectory interactions attributable to reduced power rather than a qualitative change in the pattern of results.
